# Electrolytes and electrophysiology: what’s next?

**DOI:** 10.18632/aging.102267

**Published:** 2019-09-12

**Authors:** Raymond Noordam, William J. Young, Patricia B. Munroe

**Affiliations:** 1 Department of Internal Medicine, Section of Gerontology and Geriatrics, Leiden University Medical Center, Leiden, the Netherlands; 2 Barts Heart Centre Sr. Bartholomew’s Hospital, London, UK; 3 Clinical Pharmacology, William Harvey Research Institute, Barts and The London School of Medicine and Dentistry, Queen Mary University of London, London, UK; 4 NIHR Barts Cardiovascular Biomedical Research Centre, Queen Mary University of London, London, UK

**Keywords:** electrolytes, epidemiological research, electrophysiology, electrocardiogram

The involvement of electrolytes in electrophysiological processes in the heart has been known for over a century [1]. Electrolytes are essential in the generation of the cardiac action potential during systole, and maintenance of the cardiomyocyte refractory period during diastole. However, our knowledge of these processes is based predominantly on small studies in patients (e.g. with concurrent cardiac conditions and/or with extreme electrolyte disturbances), or from *in-vitro* experiments. The study of serum electrolyte concentrations in unselected individuals and their association with electrocardiographic parameters, may therefore provide new insights into cardiac physiology and their possible role in health and disease. This is of particular interest in the aging population which may be more prone to fluctuations in serum electrolyte concentration due to reduced physiological adaptive reserve and the effects of medication.

Usually, epidemiological studies are done in one or a few cohorts, examining an association between a certain determinant and outcome either cross-sectional or longitudinally. Testing for associations in population-based samples can be affected by different biases (e.g., selection bias, recall bias) and (residual) confounding (e.g., factors reflecting the health status of an individual). To partly overcome these limitations and to identify consistent and robust associations across populations taking into account the most important confounders, we recently published a large-scale collaborative effort (>30 cohorts; >150,000 individuals) examining the associations between the main blood electrolyte concentrations with changes on the electrocardiogram [2].

We found that low calcium and high magnesium were associated with prolongation of QT and JT intervals without significant effect on QRS duration suggesting these changes are specific to ventricular repolarization. Specifically, 2% of the population tested had calcium levels sufficiently low to prolong the QT interval by > 5ms, a cut off used by the U.S Food and Drug Administration (FDA) as the threshold level for regulatory concern in drug development [3]. Significant prolongation of ventricular repolarization has been previously associated with increased risk of cardiovascular disease and mortality [4]. We also observed that in unselected individuals with electrolyte concentrations predominantly within normal limits, the association between potassium and ventricular repolarization was driven by the use of blood pressure medication. The reasons for this are unclear, and may be due to direct effects of these medications or could indicate the presence of concurrent medical conditions which influence repolarization reserve. This is of particular interest in older age groups who are more likely to have multiple comorbidities and a higher prevalence of polypharmacy.

Below, we highlight a number of new leads for future work that could be explored using different study methodologies:

- The influence of potassium on ventricular repolarization in cohorts taking specific medications to improve our understanding as to whether this is of clinical importance and relevance when rationalizing medication.

- Our analyses focused on unselected individuals from 30 cohorts, without specifically looking at subgroups that show a prolonged ventricular repolarization. For example, the mean age in our study was 55 years and the influence of serum electrolyte concentrations may be more pronounced in the older population. Additionally, we observed the effects of calcium on QT/JT interval durations were stronger in women than in men. Therefore, it would be of interest to investigate whether specific groups e.g. older women (or men), have stronger effects of calcium on QT/JT interval durations and what the underlying physiological basis may be - such as sex-specific factors, (common) genetic variation or the interaction with other clinical conditions.

- An important limitation of epidemiological/observational research is that causality is difficult to ascertain and associations we observed do not have a clear biological interpretation or meaning. Although electrolytes have established effects on electrophysiological processes in *in-vitro* experiments [5,6], how these translate at a population level remains uncertain. The findings in our study are of association only and should be tested for causal links. Population genetics, cellular and pharmaceutical studies, and in-depth electrocardiogram interrogation may assist in the quest to disentangle the biological background of the effects of electrolyte concentration on the electrocardiogram.

In summary, our big-data based results are not immediately applicable to clinical practice [2,7]. Nevertheless, our efforts do offer new and interesting insights which warrant further study. We invite others to use the results from our effort [2] in the quest to translate these findings to clinical practice or therapeutic interventions.
